# Roles of distinct nuclear receptors in diabetic cardiomyopathy

**DOI:** 10.3389/fphar.2024.1423124

**Published:** 2024-07-24

**Authors:** Yangyang Zheng, Yongji Xu, Li Ji, Wenqing San, Danning Shen, Qianyou Zhou, Guoliang Meng, Jiahai Shi, Yun Chen

**Affiliations:** ^1^ Department of Pharmacology, School of Pharmacy, Nantong University, Nantong, China; ^2^ School of Medicine, Nantong University, Nantong, China; ^3^ Department of Thoracic Surgery, Affiliated Hospital of Nantong University, Nantong, China

**Keywords:** DCM, nr, transcription factor, PPAR, ROR alpha

## Abstract

Diabetes mellitus induces a pathophysiological disorder known as diabetic cardiomyopathy and may eventually cause heart failure. Diabetic cardiomyopathy is manifested with systolic and diastolic contractile dysfunction along with alterations in unique cardiomyocyte proteins and diminished cardiomyocyte contraction. Multiple mechanisms contribute to the pathology of diabetic cardiomyopathy, mainly including abnormal insulin metabolism, hyperglycemia, glycotoxicity, cardiac lipotoxicity, endoplasmic reticulum stress, oxidative stress, mitochondrial dysfunction, calcium treatment damage, programmed myocardial cell death, improper Renin-Angiotensin-Aldosterone System activation, maladaptive immune modulation, coronary artery endothelial dysfunction, exocrine dysfunction, etc. There is an urgent need to investigate the exact pathogenesis of diabetic cardiomyopathy and improve the diagnosis and treatment of this disease. The nuclear receptor superfamily comprises a group of transcription factors, such as liver X receptor, retinoid X receptor, retinoic acid-related orphan receptor-α, retinoid receptor, vitamin D receptor, mineralocorticoid receptor, estrogen-related receptor, peroxisome proliferatoractivated receptor, nuclear receptor subfamily 4 group A 1(NR4A1), etc. Various studies have reported that nuclear receptors play a crucial role in cardiovascular diseases. A recently conducted work highlighted the function of the nuclear receptor superfamily in the realm of metabolic diseases and their associated complications. This review summarized the available information on several important nuclear receptors in the pathophysiology of diabetic cardiomyopathy and discussed future perspectives on the application of nuclear receptors as targets for diabetic cardiomyopathy treatment.

## 1 Introduction

**FIGURE 1:**
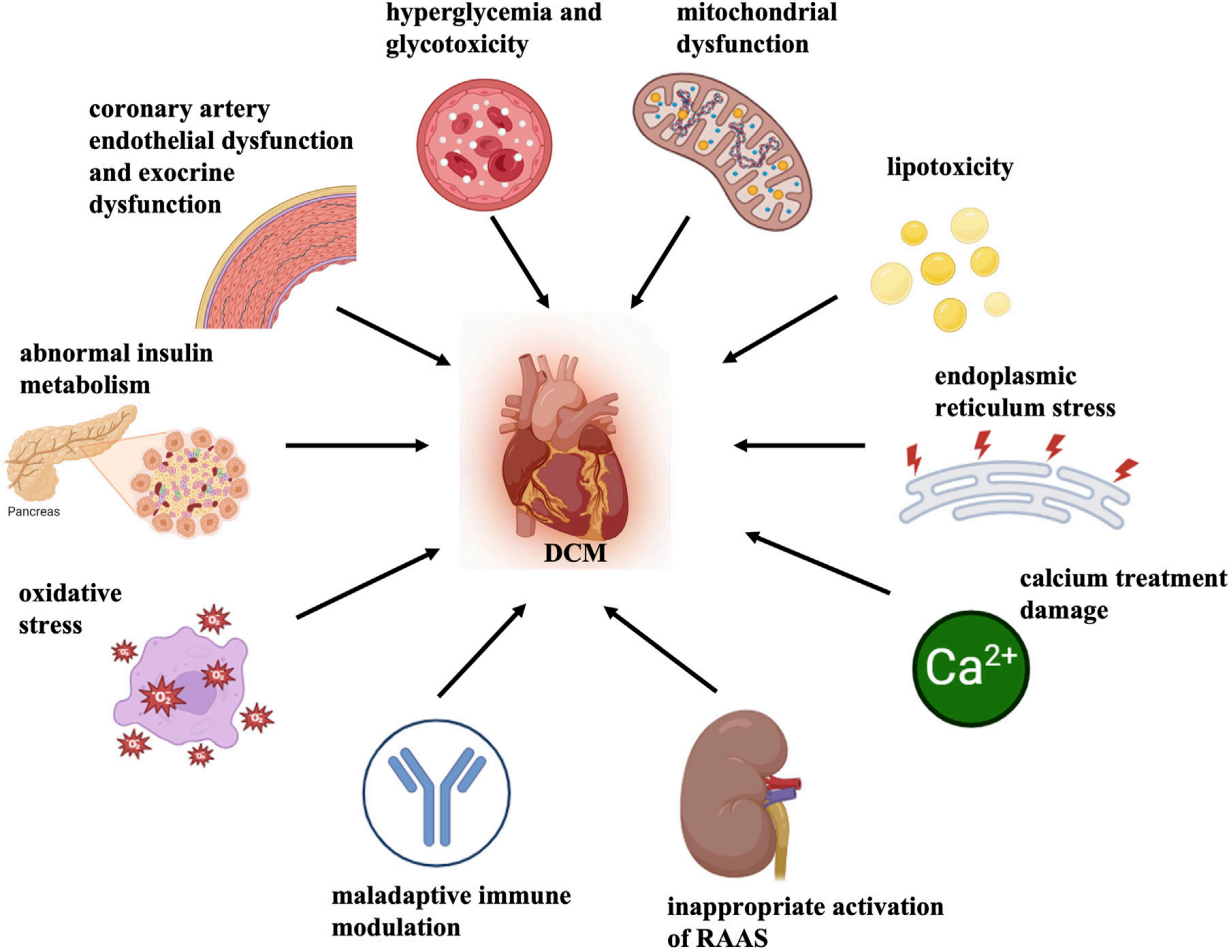
Mechanisms of DCM pathology. Multiple mechanisms contribute to the pathology of DCM, mainly including abnormal insulin metabolism, hyperglycemia and glycotoxicity, cardiac lipotoxicity, mitochondrial dysfunction and oxidative stress, endoplasmic reticulum stress, calcium treatment damage, myocardial cell death, inappropriate activation of Renin-angiotensin-aldosterone System (RAAS), maladaptive immune modulation, coronary artery endothelial dysfunction, exocrine dysfunction, etc.

Diabetic cardiomyopathy (DCM) is a specific type of cardiomyopathy that occurs in the absence of congenital heart disease, valvular heart disease, coronary artery disease, hypertension, and other cardiovascular conditions. Diabetes mellitus (DM) induces this pathophysiological disorder and may eventually cause heart failure (HF) (Madonna et al., 2023; Quaiyoom and Kumar, 2023). Systolic and diastolic contractile dysfunction along with alterations in specific cardiomyocyte proteins have been demonstrated in type 2 DM (T2D) and type 1 DM (T1D) experimental animal models (Dillmann, 2019). Multiple mechanisms contribute to the pathology of DCM, mainly including abnormal insulin metabolism, hyperglycemia, glycotoxicity, cardiac lipotoxicity, endoplasmic reticulum (ER) stress, oxidative stress, mitochondrial dysfunction, calcium overload damage and myocardial cell death, improper Renin-Angiotensin-Aldosterone System (RAAS) activation, maladaptive immune modulation, coronary artery endothelial dysfunction, and exocrine dysfunction (Jia et al., 2018; Gong et al., 2022; Li J. et al., 2023; Ao et al., 2023; Batista et al., 2023) (Figure 1). However, the exact mechanism of DCM and the diagnosis and treatment of this disease are still in the process of further exploration.

In the last few decades, various pieces of evidence have been reported that nuclear receptors (NRs) have crucial role in cardiovascular diseases. The NR superfamily consists of a group of transcription factors that are activated by a variety of chemically diverse small lipophilic ligands. These ligands include farnesol metabolites, oxidized sterols, fatty acids, thyroid hormone, 9-cis and all-trans retinoic acid, vitamin D, and sterol hormones (Libby et al., 2021; Trauner and Fuchs, 2022). The corresponding ligand binding initiates conformational alterations in the NR protein and the recruitment of co-regulators. NR is ultimately translocated into the nucleus, where it combines with the response element in the promoter region of its target gene throughout the genome, thereby regulating gene expression. The NR superfamily controls differentiation and cell growth by establishing connections between transcriptional responses and signal molecules. The NR superfamily comprises 48 members in humans, including liver X receptor (LXR), retinoid X receptors (RXR), retinoic acid-related orphan receptor-α (RORα), retinoid receptors, vitamin D receptor (VDR), mineralocorticoid receptor (MR), estrogen-related receptor (ERR), peroxisome proliferator activated receptor (PPAR), nuclear receptor subfamily 4 group A 1 (NR4A1), and others (Table 1). In recent years, the NR superfamily has received extensive attention in the field of metabolic diseases. It has been proven that they are closely related to the occurrence and development of diabetes, fatty liver, and other diseases (Hammer et al., 2021; Nemetchek et al., 2022; Walth-Hummel et al., 2022; Hu P. et al., 2023).

**TABLE 1:** The nomenclature, gene name, and ligands of human NRs.

Common name	Abbreviation	Gene names	Ligands
NR1A1	TRα	Thyroid hormone receptor-α	Thyroid hormone
NR1A2	TRβ	Thyroid hormone receptor-β	Thyroid hormone
NR1B1	RARα	Retinoic acid receptor-α	Retinoic acid
NR1B2	RARβ	Retinoic acid receptor-β	Retinoic acid
NR1B3	RARγ	Retinoic acid receptor-γ	Retinoic acid
NR1C1	PPARα	Peroxisome proliferator-activated receptor-α	Fatty acid Leukotriene B4 Phenoxyaromatic acid
NR1C2	PPARβ	Peroxisome proliferator-activated receptor-β	Fatty acid
NR1C3	PPARγ	Peroxisome proliferator-activated receptor-γ	Fatty acid Prostaglandin J2
NR1D1	Rev-ErbA	Rev-ErbAα	Not yet discovered
NR1D2	Rev-ErbAβ	Rev-ErbAβ	Not yet discovered
NR1F1	RORα	RAR-related orphan receptor-α	Cholesterol、 Cholesterol sulfate
NR1F2	RORβ	RAR-related orphan receptor-β	Retinoic acid
NR1F3	RORγ	RAR-related orphan receptor-γ	Not yet discovered
NR1H2	LXRβ	Liver X receptor-β	Oxysterols T0901317 GW3965
NR1H3	LXRα	Liver X receptor-α	Oxysterols T0901317 GW3965
NR1H4	FXRα	Farnesoid X receptor-α	Bile acid Fexaramine
NR1H5	FXRβ	Farnesoid X receptor-β	Lanosterol
NR1I1	VDR	Vitamin D receptor	Vitamin D 1,25-dihydroxyvitamin D3
NR1I2	PXR	Pregnane X receptor	Exogenous substances 16-α-Cyanopregnenolone
NR1I3	CAR	Constitutive androstane receptor	Exogenous substances Phenobarbital
NR2A1	HNF4α	Hepatocyte nuclear factor-4-α	Not yet discovered
NR2A2	HNF4γ	Hepatocyte nuclear factor-4-γ	Not yet discovered
NR2B1	RXRα	Retinoid X receptor-α	Retinoic acid
NR2B2	RXRβ	Retinoid X receptor-β	Retinoic acid
NR2B3	RXRγ	Retinoid X receptor-γ	Retinoic acid
NR2C1	TR2	Testicular receptor 2	Not yet discovered
NR2C2	TR4	Testicular receptor 4	Not yet discovered
NR2E1	TLX	Homologue of the *Drosophila* tailless gene	Not yet discovered
NR2E3	PNR	Photoreceptor cell-specific nuclear receptor	Not yet discovered
NR2F1	COUP-TFα	Chicken ovalbumin upstream promoter-transcription facto α	Not yet discovered
NR2F2	COUP-TFβ	Chicken ovalbumin upstream promoter-transcription factor β	Not yet discovered
NR2F6	EAR2	V-erbA-related	Not yet discovered
NR3A1	ERα	Estrogen receptor-α	Estradiol Tamoxifen Raloxifen
NR3A2	ERβ	Estrogen receptor-β	Estradiol
NR3B1	ERRα	Estrogen-related receptor-α	Not yet discovered
NR3B2	ERRβ	Estrogen-related receptor-β	Diethylstilbestrol 4-Hydroxytamoxifen
NR3B3	ERRγ	Estrogen-related receptor-γ	Diethylstilbestrol 4-Hydroxytamoxifen
NR3C1	GR1	Glucocorticoid receptor	Cortisone Dexamethasone RU486
NR3C2	MR	Mineralocorticoid receptor	Aldosterone Spironolactone
NR3C3	PR	Progesterone receptor	progesterone Medroxyprogesterone acetate
NR3C4	AR	Androgen receptor	Testosterone Flutamide
NR4A1	NGFIB	Nerve growth factor IB	Not yet discovered
NR4A2	NURR1	Nuclear receptor related 1	Not yet discovered
NR4A3	NOR1	Neuron-derived orphan receptor 1	Not yet discovered
NR5A1	SF1	Steroidogenic factor 1	Not yet discovered
NR5A2	LRH-1	Liver receptor homolog-1	Not yet discovered
NR6A1	GCNF	Germ cell nuclear factor	Not yet discovered
NR0B1	DAX1	Dosage-sensitive sex reversal, adrenal hypoplasia critical region, on chromosome X, gene 1	Not yet discovered
NR0B2	SHP	Small heterodimer partner	Not yet discovered

**FIGURE 2:**
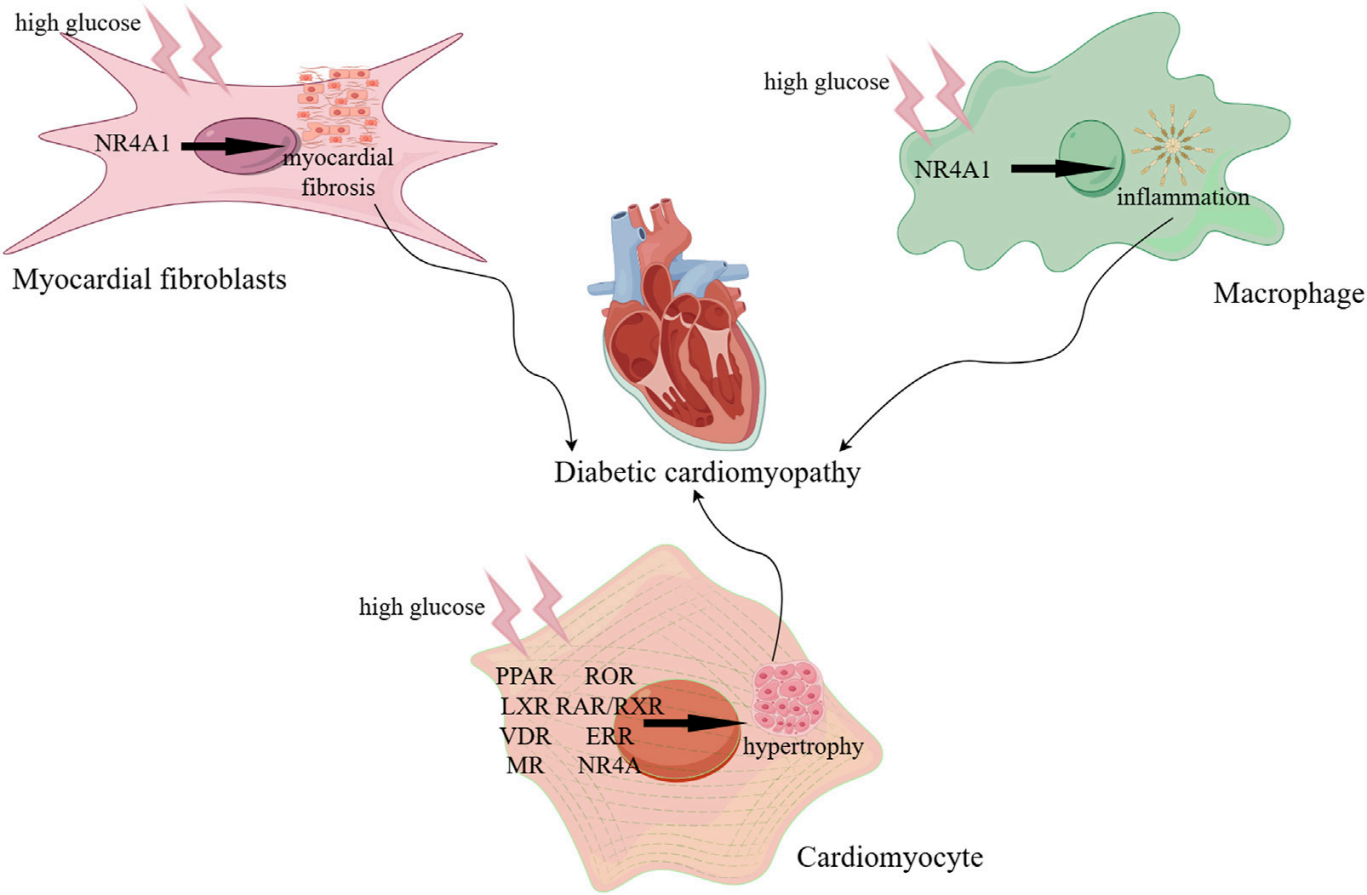
Role of myocardial fibroblasts, macrophages, and cardiomyocytes in DCM. NR4A1 functioned as an intrinsic inhibitor of diabetes-induced myocardial fibrosis. Disruption of NR4A1 contributes to high glucose-induced macrophage polarization toward a pro-inflammatory phenotype, leading to an inflammation in DCM. LXR, RXR, RORα, RAR/RXR, VDR, MR, ERR, PPAR, NR4A are involved in high glucose-induced cardiomyocyte hypertrophy in DCM.

Cardiomyocytes are the most important components of the heart and they play a central role in DCM. Cardiac hypertrophy caused by cardiomyocyte injury and dysfunction promotes the development of heart failure (Sun et al., 2023). Diabetes promotes infiltration of macrophages, along with elevated expression of inflammatory cytokine in the heart. Involvement of proinflammatory macrophage contributes to cardiomyocyte hypertrophy and myocardial fibrosis and finally leads to DCM. Besides, dysregulated macrophage polarization may contribute to DCM progression. Disruption of the NR4A1 signaling pathway was reported to be involved in macrophage polarization toward a pro-inflammatory phenotype, leading to aggravated cardiac dysfunction under diabetic conditions (Li Q. et al., 2022). Besides, cardiac fibroblast proliferation and deposition of extracellular matrix by high glucose stimulation are associated with diabetic myocardial fibrosis and ultimately resulting in DCM. NR4A1 functioned as an intrinsic inhibitor of diabetes-induced myocardial fibrosis (Figure 2) (Ma et al., 2023).

In summary, cardiac fibroblasts and macrophages dynamically interact with cardiomyocytes through mechanical, chemical, and electrical signaling in the etiology and progression of DCM. Nuclear receptors play a crucial role in this process by regulating gene transcription, affecting cell metabolism, growth, differentiation, and immune regulation. The above-stated receptors are likewise termed as metabolic nuclear receptors. In this review, the role of NRs in DCM and its mechanism of action are discussed.

### 1.1 Peroxisome proliferator activated receptors

**FIGURE 3:**
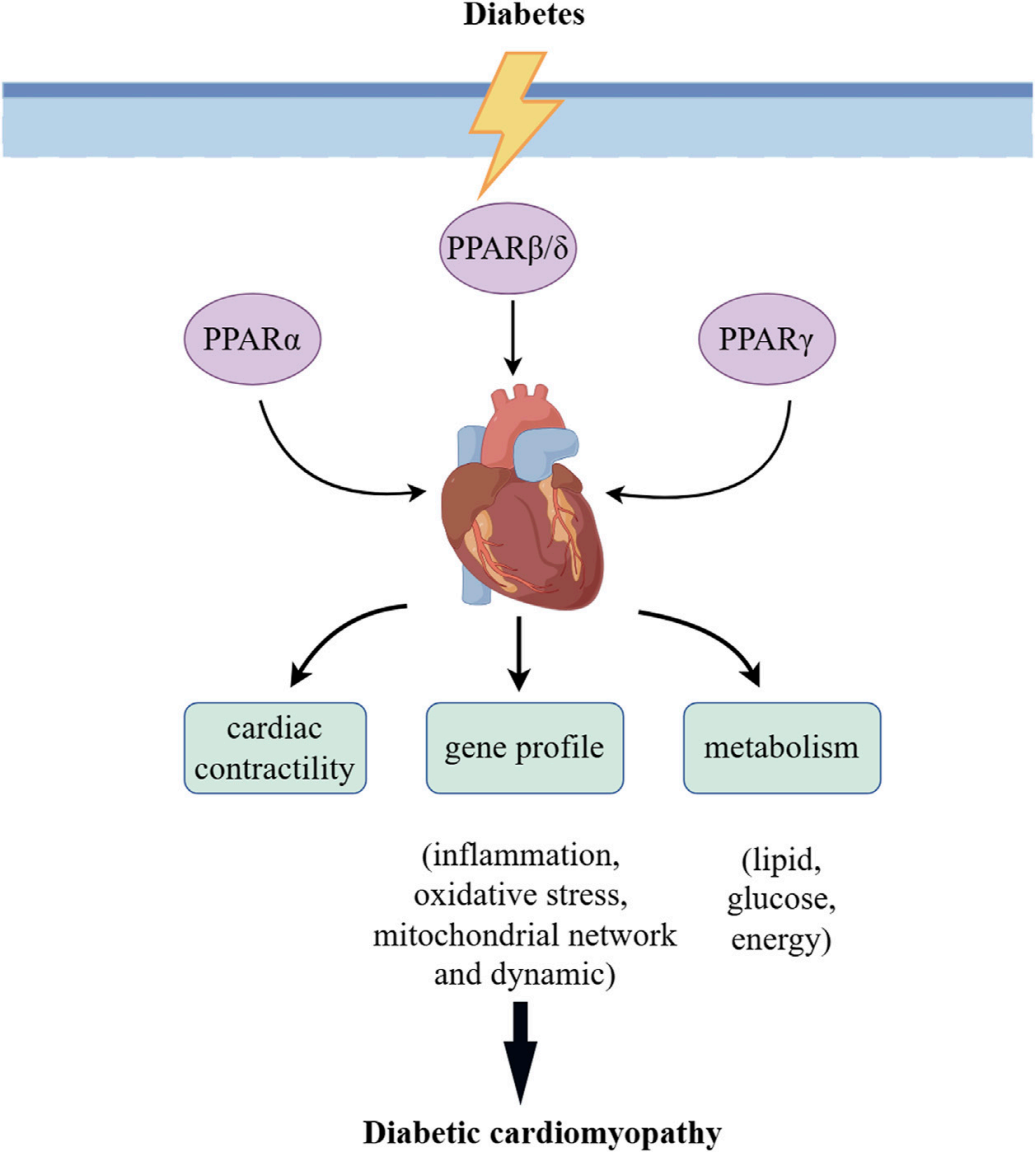
PPAR in DCM. PPARs have a significant effect on the regulation of cardiac contractility and modulation of gene profiles; they are involved in inflammation, mitochondrial network and dynamics, oxidative stress, and the modulation on metabolism regarding whole-body energy metabolism, modulation on metabolism, such as lipid, glucose, and energy.

PPARs being the subfamily of transcription factors/ligand-activated nuclear receptors belong to the nuclear hormone receptor superfamily (Michalik et al., 2006). The PPAR subfamily contains three distinct subtypes in humans and rodents. They are designated as PPARγ (NR1C3), PPARβ/δ (NR1C2), and PPARα (NR1C1), which are coded by independent genes and have distinctive tissue distribution, ligand specificity properties, and metabolic regulatory activities (Han et al., 2017). PPARs have a typical structure of nuclear receptors composed of several domains, including carboxyl terminal ligand-binding domain (LBD), central zinc finger DNA-binding domain (DBD), amino-terminal domain, as well as a small hinge area connecting LBD and DBD ((Wang, 2010)). Amino-terminal domain activates and determines the specificity of the target gene, while the LBD participates in the interaction with its necessary heterodimer partner, the retinoid X receptor and other co-regulators. PPARs have a significant effect on the regulation of cardiac contractility and the modulation of gene profiles; they are involved in inflammation, mitochondrial network and dynamics, oxidative stress, and modulation on metabolism, such as energy balance, blood glucose levels, adipogenesis and lipid synthesis, oxidative phosphorylation, esterification, storage, and transport (Figure 3) (Mao Z. et al., 2021; Wagner and Wagner, 2023; Titus et al., 2024).

#### 1.1.1 Effects of PPAR receptor on cardiac contractility

Activation of PPARs is reported to play a prominent role in the regulation of cardiac contractility. A significant decrease of PPARδ expression was detected in the hearts of STZ-rats (Yu et al., 2008). Cardiac deletion of PPARδ resulted in reduced contraction and decreased cardiac output (Cheng et al., 2004). Cardiac agents, such as digoxin and dobutamine, which improved cardiac contractility in diabetic rats, were mainly associated with elevation of cardiac PPARδ (Chen et al., 2011; Chou et al., 2012). Cheng et al. found that the activation of PPARδ was responsible for the restoration of cardiac failure in STZ-rats by ginseng (Tsai et al., 2014). Pharmacological blockade of PPARδ inhibited the improvement of cardiac performance by Ginsenoside Rh2 (Rh2) in STZ-diabetic rats (Lo et al., 2017). However, treatment with PPARγ agonist, 2-(2-(4-phenoxy-2-propylphenoxy) ethyl) indole-5-acetic acid (COOH) improved cardiac metabolism but not contractile function in type 2 diabetic db/db mice (Carley et al., 2004). Therefore, further clarification is needed to illustrate the effectiveness of PPAR activation against cardiac contractility in DCM.

#### 1.1.2 Effects of PPAR receptor modulation on genetic profile

PPARs are involved in the expression of key genes that participate in inflammation (such as nuclear factor kappa-B, NF-κB), mitochondrial network and dynamics (such as Mitofusins, Mfn 2), and oxidative stress (such as NADPH oxidase 4, NOX4) in the cardiovascular system.

A recent study revealed that ubiquitin-specific protease 28 (USP28) served as the suppressor of myocardial dysfunction and mitochondrial morphofunctional deficits in DCM. Mechanistically, USP28 directly interacted with PPARα, deubiquitinated and promoted Mfn2 transcription by stabilizing PPARα (Lys152), finally blocking the morphofunctional defects of mitochondria (Xie et al., 2024). Bioinformatics analysis reveals that PPARα activates the differentially expressed gene 3-hydroxy-3-methylglutaryl coenzyme A synthetase 2 (HMGCS2) in DCM. The existing research confirms that silencing PPARα could mitigate cardiomyocyte injury and oxidative stress through a mechanism associated with the downregulation of HMGCS2. A previous study has shown that the reduced myocardial inflammatory response through downregulating osteopontin (OPN) expression by PPAR‐γ protected against DCM(32). Li et al. reported that Krüppel-like Factor 9 (KLF9) aggravated myocardial dysfunction, the inflammation and oxidative stress response in mice with diabetic cardiomyopathy by inhibiting PPARγ/nuclear respiratory factor 2 (NRF2) signaling (Zhu et al., 2023). PPARγ bound to the PPAR response element (PPRE) in the promoter region of mitochondrial ketogenic enzymes HMGCS2, pyruvate dehydrogenase kinase isoform 4 (PDK4), and β-hydroxy butyrate dehydrogenase (BDH1), modifying their expression. This process was associated with mitochondrial dysfunction and contributed to cardiac toxicity in diabetes (Caglayan et al., 2008).

PPARs can induce or suppress the transcription of target genes. Although many protein‐coding genes have been shown to mediate the downstream effects of PPARs, the pleiotropic benefits of PPARs may exceed their effects on the modulation of genetic profiles.

#### 1.1.3 Effects of PPAR receptor modulation on metabolism

PPARs are the master regulators in cardiomyocyte metabolism. PPARα regulates gene transcription with a role in the oxidation of fatty acid (like peroxisome proliferator activated receptor gamma coactivator-1α, PGC1α), fatty acid uptake (such as cluster of differentiation 36, CD36), and glucose metabolism (such as pyruvate dehydrogenase kinase 4, PDK4) (Bekhite et al., 2021; Jayakumari et al., 2021; Kyriazis et al., 2021; Hu T. et al., 2023; Zhu et al., 2023; Xie et al., 2024). Cardiac muscles have a higher expression of PPARβ/δ similar to that of PPARα and is the master regulator of myocardial lipid metabolism (Cabrera et al., 2021; Bauersachs and Lopez-Andres, 2022). PPARα is also expressed in tissues that undertake pivotal catabolism of fatty acid, like intestine, kidney, liver, and brown adipose tissue (Xia et al., 2023). The energy regulation sensitivity of diabetic patients decreases and the expression of intracellular glucose transporters (GLUT1 and GLUT4) is reduced. This reduction leads to the upregulation of PPARα, further increasing the expression of fatty acid degrading enzymes and causing insulin resistance. PPARβ/δ regulates both fatty acid oxidation (FAO) and gene activation involved in glucose transport and glycolysis in the cardiomyocytes (Huang L. et al., 2020). While less abundance of PPARγ is displayed in the heart, its activation induces changes in lipid and glucose utilization, manages hyperglycemia, and exerts additional beneficial effects (Montaigne et al., 2021; Wu et al., 2022; Pham et al., 2023). Early studies have shown that overexpression of Mitsugumin 53 (MG53) induced the mRNA levels of PPARα and its target genes. PPARα inhibition by gene silencing attenuated the MG53-induced lipid uptake in cardiomyocytes, leading to lipid accumulation and toxicity, thereby resulting in DCM (Liu et al., 2015). Huang and colleagues found that diabetic mice displayed marked myocardial fatty acids uptake and oxidation, as evidenced by increased mRNA expression of myocardial fatty acids uptake and oxidation embracing CD36, fatty acid transporter 1 (FATP1), carnitine palmityl transferase 1 (CPT-1), fatty acyl coenzyme A synthetases (FACS), and medium-chain acyl-CoA dehydrogenase (MCAD). However, there was decreased fatty acids uptake and oxidation, along with reduced expression of glucose transporter, GLUT4/GLUT1, in diabetic mice after severe hypoglycemia (SH) delivery. Interestingly, key transcriptional regulators of fatty acid metabolism, PPARα, PPARβ/δ, and PPARγ were all increased in diabetic mice but there was reduced PPARβ/δ and PPARγ in diabetic mice after SH delivery. The above results suggested that SH inhibited myocardial metabolism related to PPARβ/δ in diabetic mice (Huang L. et al., 2020). PPARγ overexpression was accompanied by intramyocardial lipid accumulation in the samples of left ventricular biopsy from patients with metabolic syndrome, which may lead to DCM (Marfella et al., 2009). Till now, a number of drugs and compounds protect against DCM via activating PPARγ, such as the combination of dapagliflozin, *Lactobacillus*, and crocin; pioglitazone and curcumin; and resveratrol, the Chinese medication qiliqiangxin, polysulfide, ginsenoside Rg3 (an extract from Panax notoginseng) (Gbr et al., 2021; Wu et al., 2021; Fang et al., 2022; Khalaf et al., 2022; Xiong et al., 2023).

Conjointly, these findings clarify that PPARs may serve as the potential target in treating DCM.

## Estrogen-related receptor

ERR is an orphan nuclear receptor belonging to the NR superfamily with three subtypes, including ERRγ, ERRβ, and ERRα. ERRs possess structural features typical of NRs, including an activation function (AF)-1 domain, a DBD, a LBD, and an AF-2 domain. Emerging evidence has convinced that the ERR family functions as PGC-1-activated regulators in cardiac energy metabolism process (Sopariwala D. et al., 2023; Cao et al., 2023). Multiple research studies reveal that ERRs have a role in the occurrence and development of cardiovascular diseases like peripheral arterial disease and critical limb ischemia, calcific aortic valve disease, cardiac hypertrophy, and heart failure (Hu et al., 2011; Kwon et al., 2013; Hu et al., 2021; Sopariwala DH. et al., 2023). ERRγ was enhanced in db/db mice heart by screening for potential regulators of different gene expressions in diabetic mice. ERRγ overexpression by transfecting adenovirus into cultured cardiomyocytes was adequate to trigger cardiomyocyte hypertrophy, enhance palmitate oxidation, and increase gene expression included in lipid oxidation (Lasheras et al., 2016). Ma et al. found that the activation of silent information regulator 1 (SIRT1) by resveratrol alleviated myocardial damages in DCM through PGC-1α, NRF1, NRF2, ERR-α, and mitochondrial transcription factor A (TFAM)-mediated mitochondrial regulation (Ma et al., 2017).

Development of some ERR knockout and overexpression models along with the employment of advanced functional genomics may facilitate the understanding of ERR pathways in DCM.

## 3 Mineralocorticoid receptor

Aldosterone is a mineralocorticoid steroid hormone that exerts its classic effects by activating the mineralocorticoid receptor (MR), which is widely expressed in the adipose tissue, central nervous system, heart, colon, and kidney (Barrera-Chimal et al., 2022). The aldosterone-MR complex has a dominant function in regulating blood pressure and extracellular volume homeostasis and in the control of serum potassium levels. In addition, the aldosterone-MR complex may directly stimulate the proliferation of cardiomyocytes and fibroblasts in response to inflammation or damage (Bauersachs and Lopez-Andres, 2022). Classically, aldosterone binds to cytosolic MRs and subsequently translocates to the nucleus to regulate gene transcription and translation of proteins, such as serum- and glucocorticoid-induced protein kinase 1 (SGK1) (Cabrera et al., 2021). Besides, aldosterone displays non-genomic effects through the activation of extracellular receptor kinase, for instance, Rho kinase and protein kinase C, which mediate cardiovascular tissue remodeling (Lu et al., 2019). As the heart does not express 11-beta-hydroxysteroid dehydrogenase 2 (11β-HSD2) that converts endogenous glucocorticoids to receptor-inactive 11-keto analogues, both aldosterone and glucocorticoids can activate the MR in the heart. The effects of glucocorticoids are adjusted via the cells’ redox state and they stimulate MR signaling when it is under an oxidized state (Morales et al., 2022).

Given the influence of improper activation of RAAS in the pathophysiology of DCM, inhibition of MR activation appears to be an appropriate therapeutic strategy. Early studies have shown that by blocking MR, eplerenone mitigated cardiac steatosis and myocardial apoptosis as well as later cardiac remodeling and diastolic dysfunction in obese/type II diabetic rats (Ramirez et al., 2013). In AT1aR KO (angiotensin II type 1a receptor knockout diabetic mice model), RAAS blockade alleviated left ventricle dysfunction, apoptosis of cardiomyocyte, and augmented oxidative stress 6 weeks after STZ injection but not after 12 weeks. Interestingly, the expression of MR mRNA had no increase in AT1aR KO diabetic mice at 6 weeks, however, it has been upregulated at 12 weeks. Further interruption of MR signaling in AT1aR KO diabetic mice with eplerenone prevented left ventricle dysfunction, myocardial apoptosis, and augmented oxidative stress in diabetic mice at 12 weeks after STZ injection (Nagatomo et al., 2014). This study indicated that AT1 repression alone protected heart damage through diabetes in short term. However, inhibiting MR and AT1 signaling in the long-term is essential. A study using RNA sequencing analysis and network pharmacology methods reported that asaxerenone, a new non-steroidal MR antagonist, targets the chemokine and phosphatidylinositol 3-kinase (PI3K)-protein kinase B (Akt) signaling pathway to facilitate therapeutic effects on DCM (Li Z. et al., 2023). *In vivo* study found that a third-generation mineralocorticoid receptor antagonist finerenone attenuated cardiac dysfunction, cardiac hypertrophy, cardiac fibrosis, and apoptosis of cardiomyocyte in Zucker Diabetic Fatty (ZDF) rats. *In vitro* studies have shown that finerenone regulated lipid metabolism through the PPARγ/CD36 pathway and reduced high glucose and high fatty acid stimulated cardiomyocytes apoptosis and mitochondrial dysfunction via the caspase 8/tumor necrosis factor receptor (TNFR1)/tumor necrosis factor α (TNFα) pathway (Jin et al., 2023). Further research is warranted to better enable the development of a clinically efficacious strategy for managing MR in patients with DCM.

## 4 Vitamin D receptor

Essentially, VDR is a ligand-dependent nuclear transcription factor that regulates calcium and phosphorus metabolism. It also has a function in mediating the cell differentiation and proliferation by binding to the vitamin D active metabolite (Dillmann, 2019), i.e., 1,25-dihydroxyvitamin D3 (1,25D3, calcitriol). Studies have revealed that, in addition to calcitriol, lithocholic acid, curcumin, polyunsaturated fatty acids, and gamma-tocotrienol can also activate VDR (Latic and Erben, 2022; Guo et al., 2022). VDR has a modular organization that includes flexible, variable, and short N-terminal domain, conservative LBD, highly conserved DBD, and a hinge area that interlinks LBD to the DBD. Furthermore, VDR possesses distinctive character regarding NRs, having long insertion area in LBD that remains in a disordered form ((Rochel, 2022)). There are two types of VDR: the nuclear receptor (nVDR) and the cell membrane receptor (mVDR) with a molecular weight of 50 KDa and 60 KDa, respectively (Zhang Y. et al., 2023).

In the observational studies, it was found that the activation of VDR is associated with a reduced cardiovascular risk and enhanced survival. In a model of STZ-induced T1DM rats, 1,25-dihydroxyvitamin D3 partially attenuated interstitial fibrosis and cardiac hypertrophy, attenuated cardiac dysfunction, and recovered diabetic rats’ impaired cardiac autophagy. However, the aforementioned effects were nullified when the endogenous cardiac VDR gene was blocked in the rats with diabetes. Incubation with 1,25D3 in high glucose (HG)-stimulated H9C2 cells increased the VDR expression and enhanced autophagy formation by inhibiting the glycogen synthase kinase-3β (GSK-3β)/T-cell factor/lymphoid enhancer factor (TCF4)/β-catenin/mammalian target of rapamycin (mTOR) pathway (Wei et al., 2017). Furthermore, they also demonstrated VD-VDR signaling provided protection against DCM partially through the Sirtuin 1 (SIRT1)/poly (ADP-ribose) polymerase 1 (PARP1)/mTOR pathway (Qu et al., 2017). Recent studies have consistently reported that 1,25D treatment ameliorated myocardial autophagy and injury via VDR activation for inhibiting the nuclear translocation of Forkhead box protein O1 (FoxO1) in Zucker Diabetic Fatty (ZDF, fa/fa) rats. VDR ablation with small interfering RNA (siRNA) in high glucose-exposed cells indicated that autophagy regulation and the following death of cells in diabetic cardiomyocytes through 1,25D is linked with the activity of VDR ((Guo et al., 2020)). All these data demonstrate the vital role of VDR in the treatment of DCM with 1,25D3 supplementation, highlighting and offering a conducive methodology to prevent and treat DCM.

## 5 Retinoid receptors

Retinol (vitamin A) produces retinoic acid (RA) as an active metabolite that participates in the regulation of cell differentiation and proliferation. The pleiotropic activities of RA are mediated by the two nuclear receptor types, i.e., retinoid X receptor (RXR) and retinoic acid receptor (RAR). Both RAR and RXR have three subtypes, γ, β, and α. RXRs combine with stereoisomer, 9-cis-RA, while RARs mainly bind with all-trans retinoic acid (ATRA). Then, the dimers are formed by receptors that bind with DNA motifs called RA response elements (RAREs) present in the regulatory areas of targeted genes. Afterward, these regulate the transcription of a variety of targeted genes (Schubert and Germain, 2023). RXR regulates the transcription of genes via the formation of heterodimers or homodimers with many nuclear receptors, such as thyroid hormone receptor, LXR, farnesoid X receptors, PPARs, VDR, and RAR ((Shao et al., 2021)). Both RXR and RAR possess conserved structures consisting of six areas. N-terminal possesses AF-1 transcriptional domain that functions autonomously and independently of ligands. The most conserved DBD is in the central region, while C-terminal has LBD and ligand-dependent AF-2 ((Liang et al., 2021)).

Evidences have confirmed alterations in the RA signal pathway either through changes in intracellular or extracellular RA levels or RAR/RXR expression associated with DCM. Guleria et al. reported downregulated nuclear RXRα and RARα, apoptotic signaling activation, and apoptosis of cells in cardiomyocytes exposed to HG and in hearts of diabetic ZDF rats, contributing to diabetic cardiac remodeling. The use of both RAR and RXR agonists suppressed reactive oxygen species (ROS) production and HG-stimulated apoptosis. Besides, RARα and RXRα silencing by siRNA promoted HG-stimulated apoptosis and RAS components expression. RAR and RXR activation with ATRA pretreatment inhibited the impacts of hyperglycemia on RAS components expression, ROS generation, and cell apoptosis (Guleria et al., 2011). Collectively, RAR/RXR signaling activation shows cardioprotective impact by inhibiting RAS components’ cardiac expression and attenuating hyperglycemia and oxidative stress-induced apoptosis. This represents a novel therapeutic target for development in treating DCM. Moreover, LGD1069 (RXR agonist) and Am580 (RARα agonist) reduced oxidative stress, promoted glucose utilization and protein kinase B (Akt) activation, improved insulin resistance, and promoted diabetes-induced cardiac dysfunction and pathological changes via enhancing glucose tolerance. These effects were achieved by modulating NF-κB signaling and interrelated mitogen-activated protein (MAP) kinase pathways (Guleria et al., 2013). A recent study suggested that the RXR agonist bexarotene (Bex) attenuated DCM by inhibiting cardiac fibrosis through the activation of signaling by liver kinase B1 (LKB1) and the inhibition of p70S6K (p70 ribosomal protein S6 kinase) in STZ-induced rats (Chai et al., 2020). Thus, analyzing RXR and RAR molecular mechanisms in regulating DCM would be of great significance for prospective strategies in the improvement of efficacious treatments for DCM.

## 6 Retinoic acid-related orphan receptor-α

RORs have three members, i.e., RORγ, RORβ, and RORα, encoded with independent genes and exhibit definite cell- and tissue-specific expressions. RORα is ubiquitously distributed in the heart, skeletal muscle, lung, liver, brain, and cerebellum. RORβ is mostly been expressed in the central nervous system. RORγ predominantly exists in the thymus (Chen et al., 2023). Here, we discuss the most characterized RORα in regulating DCM. RORα like other NRs are functionally and structurally categorized into various regions, including the N-terminal domain, the DBD, and the LBD. Then multiple downstream events are evoked based on the binding of specific ligands by LBD. RORα controls gene transcription and expression by binding to ROR response element (RORE) (Xiong and Xu, 2022). Observations have confirmed that RORα plays diverse roles in lipid metabolism, immunoregulatory, redox homeostasis regulation, circadian rhythm regulation, and anticancer (Huang H. et al., 2020; Mao W. et al., 2021; Ferreira et al., 2021; Hams et al., 2021).

**FIGURE 4:**
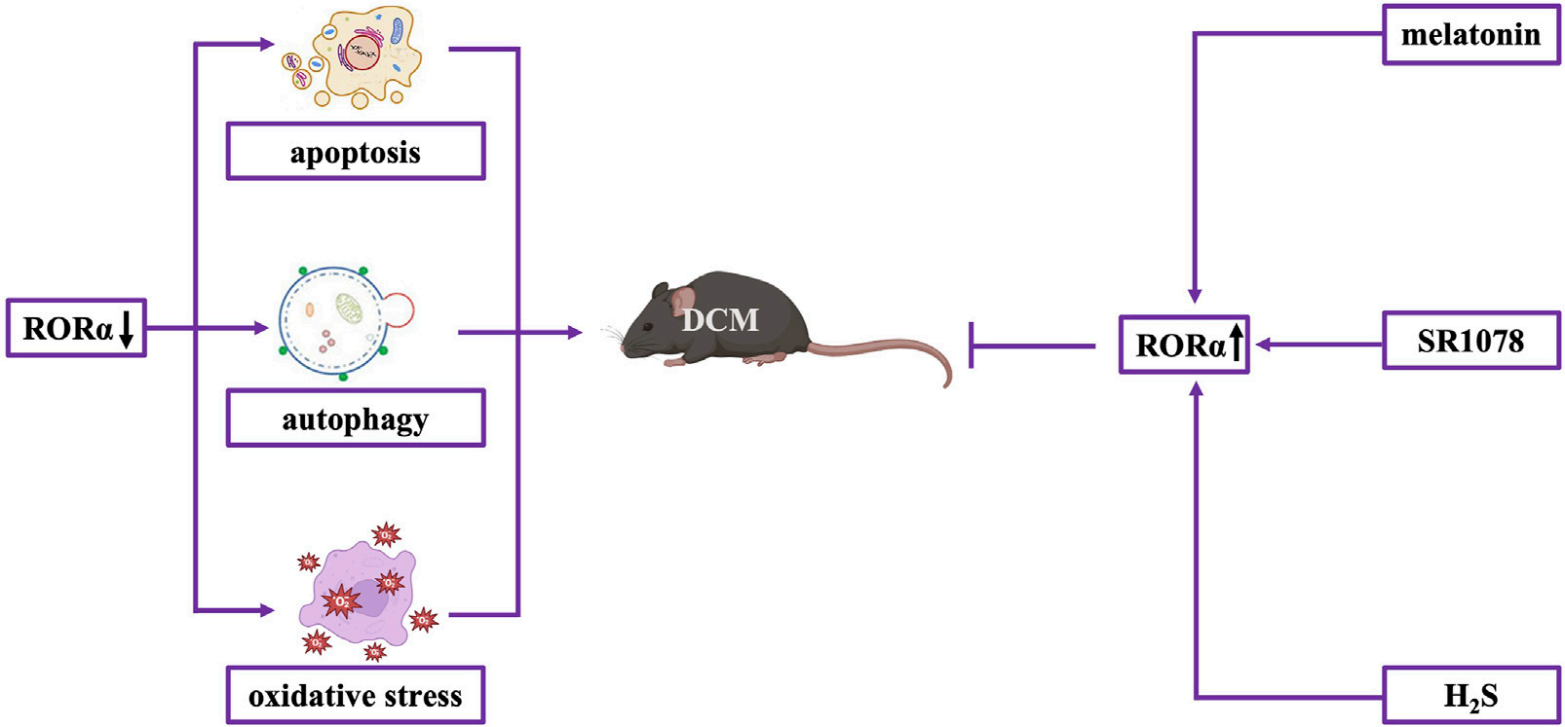
RORα in DCM. RORα is markedly reduced in diabetic hearts. RORα deficiency exacerbates myocardial apoptosis, autophagy dysfunction-mediated cell death, and oxidative stress. RORα activation by melatonin, SR1078, and H_2_S supplement protects against DCM.

It is noteworthy that RORα plays crucial roles in DCM. Melatonin, an endogenous substance, is identified as a natural agonist of RORα. Under high-glucose conditions, RORα serves as the primary receptor responsible for the cardioprotective benefits of melatonin, which include proautophagic, antioxidative, and antiapoptotic effects. For example, Zhao et al. found that RORα was significantly downregulated in diabetic hearts and in cardiomyocytes under high-glucose conditions. This downregulation aggravated myocardial diastolic dysfunction and cardiac remodeling. By employing mouse line having RORα deletion, researchers exhibited that deficiency of RORα deteriorated diastolic function and augmented diabetes-induced cardiac remodeling. Mechanically, RORα deficiency exacerbated myocardial apoptosis, oxidative stress and dysregulated-autophagy-mediated cell death. In comparison, transgenic diabetic mice exhibiting restoration of cardiac RORα levels showed improvements in cardiac functional and structural parameters. Consistently, RORα activation by SR1078 (the synthetic agonist of RORα) and melatonin protected for DCM, whereas RORα inhibitor SR3335 markedly aggravated myocardial dysfunction in diabetic mice (Zhao et al., 2017). Our recent study found that exogenous hydrogen sulfide (H_2_S) supplementation caused phosphorylation of signal transducer and activator of transcription 3 (STAT3) at Ser727, suppressed oxidative stress, decreased necroptosis, and ameliorated DCM through a RORα-dependent mechanism (Zhang S. et al., 2023) (Figure 4). In summary, there is assertive evidence for cardioprotection against DCM by RORα, although clear mechanistic details are still pending to be elucidated.

## 7 Liver X receptors

LXRs have two subtypes, i.e., LXRβ and LXRα. Target gene transcription is activated by them via the interaction with LXR response element (LXRE) and through heterodimerizing with RXR. They can sense cholesterol homeostasis and are indispensable to protect in cases of cardiovascular disorders. LXRs also play crucial regulatory roles in glucose metabolism, insulin synthesis, and secretion. In early studies, researchers identified LXR as a novel tool for regulating gene expression, such as GLUT4, which is reduced in conditions of diabetes and insulin resistance (Dalen et al., 2003). Activation of LXR by AZ876 inhibited the increase of genes related to hypertrophy and fibrosis, further suppressing pro-hypertrophic and pro-fibrotic signaling. It also reduced the increases in heart weight induced by transverse aortic constriction as well as myocardial fibrosis and cardiac dysfunction (Cannon et al., 2015). Given that the robust anti-diabetic and cardioprotective capabilities of LXRs have been extensively studied, the role of LXRs in DCM is increasingly intriguing. For example, LXRα expression in the right and left ventricles and atria of STZ-induced diabetic rats enhanced during the period of DCM progression (Cheng et al., 2011). Additionally, LXRα activation through GW3965 markedly prevented apoptosis by regulating miR-1 and mitochondrial pathway of HG-induced H9C2 cells (Cheng et al., 2018). Consistently, GW3965 improved mitochondrial fusion, reduced the expression of Calpain1, inhibited mitochondrial fragmentation and fission, and in turn promoted functions of mitochondria, and suppressed apoptosis in cardiomyocytes under high-glucose conditions. Furthermore, the mitoprotective capability of LXR activation was abrogated in LXRα however not in LXRβ-knockdown cardiomyoblasts (Lin et al., 2022). Cardiac-specific overexpression of LXRα in type 2 diabetes mouse model as triggered by a high-fat diet (HFD), alleviated the progress of HFD-induced left ventricular hypertrophy. This is associated with enhanced natriuretic peptide signaling and glucose dependence during the early stage of DCM (Cannon et al., 2016). Therefore, targeting LXRα may hold promise for the development of DCM therapies.

## 8 Nuclear receptor subfamily 4 group A

NR4A comprises three members: NR4A1, NR4A2, and NR4A3, which are formerly annotated as Nur77, Nurr1, and NOR1, respectively. Structurally, the NR4A family comprises an N-terminal transactivation domain, a central DBD, and a C-terminal putative LBD. They are orphan receptors due to their lack of endogenous ligands and function as transcription factors or inhibitors by binding to NGFI-B- or Nur-response elements (NBRE or NurRE) in the DNA sequence. These elements are typically in the promoter regions of genes. Additionally, the NR4As exert non-genomic activities, interacting with the activity, stability, and degradation of many other transcription factors and co-regulatory proteins via the NTD and LBD (He et al., 2023; Wang et al., 2023).

NR4A1, also known as Nur77, is mostly reported in the heart and plays a vital role in the regulation of glucose and lipid metabolism, inflammation, and vascular homeostasis. Studies have reported that NR4A1 exhibits antifibrotic effects by inhibiting transforming growth factor-beta (TGF-β) signaling. Ma et al. illustrated the protective effect of cytosporone B (Csn-B) on fibrosis in cardiac fibroblasts and diabetic mouse models by activating NR4A1 expression. NR4A1 recruits TGF-β target genes, SIN3A, and histone deacetylase 1 (HDAC1) to form a repressor complex, countering the TGF-β-mediated pro-fibrotic effects in diabetes (Ma et al., 2023). Another study found that the absence of lipocalin 10 (Lcn10) disrupted the nuclear translocation of NR4A1 in macrophages, leading to limited NR4A1 activation. This disruption exacerbated the inflammatory response and the accumulation of pro- and anti-inflammatory macrophages in the heart during diabetes, ultimately worsening cardiac dysfunction (Li Q. et al., 2022). Surgical bariatric procedures, such as sleeve gastrectomy (SG), alleviated the pathological cardiac hypertrophy, myocardial fibrosis, and myocardial contraction and dysfunction in myocardial contraction and diastole of a rat model of DCM induced by a high-fat diet and low-dose STZ administration. Mechanically, SG surgery activated the adenosine monophosphate activated protein kinase (AMPK) signaling, reduced NR4A1 levels, improved mitochondrial dysfunction, and enhanced myocardial energy production (Li S. et al., 2022). Wang et al. found that treatment with an FGF1 variant (FGF1^∆HBS^) activated AMPK, decreased NR4A1, thereby restoring mitochondrial function and protecting against myocardial remodeling and dysfunction in diabetes (Wang et al., 2021).

NR4A2 dysfunction is also involved in DCM. Researchers found low expression of NR4A2 in rats with DCM. Overexpression of NR4A2 promoted M2 polarization of macrophages, alleviated cardiomyocytes loss, attenuated myocardial injury, and fibrosis by inhibiting the transcription of C-C chemokine receptor 5 (CCR5) in cardiac tissues of diabetic rats (Miao et al., 2022).

In summary, NR4A nuclear receptors are emerging as key players in cardiac stress response and thus may be novel therapeutic targets in DCM.

## 9 Conclusion and future prospects

There is currently no specific pharmacotherapy available for dilated DCM, either based on NRs or any other pharmaceutical approach. Current treatments mainly focus on lowering the blood glucose and blood pressure to prevent heart failure. A substantial amount of evidence has clearly verified that regulating the NRs as discussed in this review contributes to retarding or even preventing DCM. A large number of pharmacological methods specifically targeting these NRs have been proven beneficial in suspending myocardial dysfunction in preclinical research studies or clinical trials. Some NR agonists and antagonists have been successfully used for the treatment of DCM.

There are some limitations too. For example, there are no genomic mapping studies of NRs in DCM, as NR binding profiles are cell-specific and tissue-specific. Besides, multiple NRs co-exist in the heart, and the crosstalk among NRs needs to be clarified during DCM.
